# The relationship between cannabis use and taurine: A MRS and metabolomics study

**DOI:** 10.1371/journal.pone.0269280

**Published:** 2022-06-02

**Authors:** Sharlene D. Newman, Ashley M. Schnakenberg Martin, David Raymond, Hu Cheng, Landon Wilson, Stephen Barnes, Brian F. O’Donnell

**Affiliations:** 1 Alabama Life Research Institute, The University of Alabama, Tuscaloosa, Alabama, United States of America; 2 Department of Psychiatry, Yale University School of Medicine, New Haven, Connecticut, United States of America; 3 Psychology Service, VA Connecticut Healthcare System, West Haven, Connecticut, United States of America; 4 Department of Psychological and Brain Sciences, Indiana University, Bloomington, Indiana, United States of America; 5 Targeted Metabolomics and Proteomics Laboratory, University of Alabama at Birmingham, Birmingham, Alabama, United States of America; 6 Department of Pharmacology and Toxicology, University of Alabama at Birmingham, Birmingham, Alabama, United States of America; 7 Program in Neuroscience, Indiana University, Bloomington, Indiana, United States of America; Museo Storico della Fisica e Centro Studi e Ricerche Enrico Fermi, ITALY

## Abstract

Taurine is an essential amino acid. It has been shown to be neuroprotective including protecting against the neurotoxic effects of glutamate. The goal of the current study was to examine the relationship between CB use and taurine measured in brain using magnetic resonance spectroscopy (MRS), and peripherally from a urine sample. Two experiments are presented. The first is a reanalysis of published data that examined taurine and glutamate in the dorsal anterior cingulate of a CB user group and non-user group using MRS. The second experiment, in a separate CB user group, used metabolomics analysis to measure taurine levels in urine. Because body composition has been associated with the pharmacokinetics of cannabis and taurine levels, a moderation model was examined with body composition included as the covariate. The MRS study found taurine levels were correlated with glutamate in both groups and taurine was correlated with frequency of CB use in the CB user group. The moderation model demonstrated significant effects of CB use and BMI; the interaction was marginally significant with lower BMI individuals showing a positive relationship between CB use and taurine. A similar finding was observed for the urine analysis. Both CB use and weight, as well as the interaction were significant. In this case, individuals with higher weight showed an association between CB use and taurine levels. This study shows the feasibility and potential importance of examining the relationship between taurine and CB use as it may shed light on a mechanism that underlies the neuroprotective effects of CB.

## Introduction

The use of cannabis (CB) has increased over the past decade in the United States, with several states making recreational and/or medical use legal. The past-year prevalence of CB use exceeds 10% [1] with few users who become dependent seeking treatment [2]. The primary psychoactive component of CB is Δ9-tetrahydrocannabinol (THC). THC has been found to cause oxidative stress and neuroinflammation [3,4]. THC has recently been shown to interact with the NF-ĸB (nuclear factor kappa-light-chain-enhancer of activated B cells) signaling pathway [5]. The NF-ĸB signaling pathway is responsible for cytokine production, which is linked to the inflammatory response. A study by Hinckley and colleagues [6] examined the molecular pathways affected by CB use in humans via plasma. They found thirteen proteins, three metabolites and two lipids were correlated with a metabolite of THC, THC-COOH; taurine was one of those metabolites. THC has also been found to modulate glutamate levels [7,8]. Increased glutamate has been linked to increased oxidative stress and inflammation [9], which have all been linked to depression and other psychiatric disorders [10].

Taurine is abundant in the brain and is essential to brain development [11]. It is also associated with the NF-ĸB signaling pathway (see [12] for review) and modulates neuroinflammation [13]. However, unlike THC it has been shown to be neuroprotective and reduces both oxidative stress and inflammation [14]. Taurine also protects against glutamate-induced damage to neurons and inhibits N-methyl-D-aspartate receptors [15,16]. The biosynthesis of taurine occurs in astrocytes [17]. The mechanism that triggers taurine production is unclear but there is accumulating evidence that it acts to counter the neurotoxic effects of chronic activation of the NF-ĸB pathway and increases in glutamate levels. For example, in a randomized controlled trial, taurine supplementation reduced psychotic and depressive symptoms, and improved global function, in first episode patients with psychotic disorders [15]. Taurine may be a key in understanding the relationship between CB and psychological disorders. While difficult due to the spectral overlap with myo-inositol, glucose and choline [18], taurine concentrations can be measured using standard magnetic resonance spectroscopy (MRS) techniques [19,20]. To date, little attention has been given to understanding the association between taurine and CB use.

The goal of the current study is to address this gap in the literature and demonstrate an association between taurine and CB use by examining neural taurine levels in the dorsal anterior cingulate cortex (dACC) with both MRS and peripheral taurine levels by performing metabolomics analysis of urine in two separate samples of CB users. We hypothesize that CB use will engage the NF-ĸB signaling pathway which will in turn result in increases in taurine biosynthesis to reduce the deleterious effects of the chronic activation of the pathway. This effect is thought to occur both in the periphery and in the brain. We focused on the dACC as it is part of the reward system [21] that has been previously found to show differential activation [22] and functional connectivity differences [23–25] in chronic CB users. Additionally, because previous studies have linked CB use and glutamate [2,26] an association between taurine and glutamate levels is hypothesized. Because taurine is abundant throughout the body, experiment one was a reanalysis of MRS data obtained in a sample of CB users and controls [27], and experiment two examined taurine levels in urine in a different sample of CB users only.

## Methods and materials

### Experiment 1: MRS

#### Participants

MRS data from these subjects have been published previously [27]; taurine was not analyzed previously. A total of 69 current users and non-users participated in the study. Subjects were recruited by local advertisements. After detailed description of the study, written and verbal informed consent was obtained from each participant. Subjects were asked to refrain from alcohol or CB use the day prior to the MRI scan. This study was carried out in accordance with the recommendations of and approved by Indiana University’s Institutional Review Board for the protection of human subjects. All subjects gave written informed consent in accordance with the *Declaration of Helsinki*.

The exclusion criteria included: younger than 18 years or older than 40; presence of any neurological disorder; history of head trauma with loss of consciousness greater than ten minutes; learning disability; diagnosed psychological disorders including major depression, panic disorder, or psychosis; use of illicit drugs (other than CB in the user group); alcohol dependence; and contraindication to MRI. For the CB use group an additional exclusion criterion was CB use less than one instance per week. A total of 26 CB users (10 males, age 21.4±4.5, age of CB initiation 16.4±2.5 years) and 24 non-users (10 males, age 21.5±2.3).

Participants completed a battery of assessments including the Structured Clinical Interview for DSM-IV-TR (SCID-IV-TR), Research Version [28]; a written drug use questionnaire [29,30]; a six-month timeline follow back assessment to estimate current and past use of CB and alcohol; the short Michigan alcohol screening test (SMAST); and the Wechsler Abbreviated Scale of Intelligence (WASI [31]). The control subjects had no history of substance dependence, a negative urine screen for CB and other substances, and no use of CB in the past three months. Groups did not significantly differ in age, IQ score, sex, days since last alcohol use or drinks per week at the time (*p’s* > 0.1). Additionally, when examining just the CB group, there were no sex differences in age, age of CB use onset, monthly CB use, or lifetime CB use (*p’s* > 0.1). Cannabis use disorder was not a requirement for the CB user group (see 20 for demographic details).

### MRS analysis

Image acquisition information can be found in Newman et al. [27].

The MRS data were processed with LCModel (http://www.s-provencher.com/, version 6.2-0R) using default settings for water attenuation, estimated water concentration and baseline modeling. LCModel fits each spectrum as a weighted linear combination of a basis set of in vitro spectra from individual metabolite solutions. The basis set was provided by LCModel for TE 30 ms and 123 MHz. The water reference signal was used for eddy current correction and scaling the metabolite concentrations. The concentrations of taurine and glutamate were expressed in institutional units. LCModel also reports an estimated relative standard deviation (%SD) for each fitted component, which is equivalent to the Crame´r-Rao lower bounds (CRLB). Subjects were excluded if the sum of CRLB values of creatine and phosphacreatine was greater than 20%. The mean %SD for taurine was not significantly different between groups [p>0.9; CB user group: 24.5%; non-user: 24.3%]. The absolute neurometabolite concentrations were obtained using a method described by Gussew and colleagues [32]. Additional parameters for the correction included the T1 and T2 relaxation time of water in GM (1.82/0.10 s), WM (1.08/0.07 s), and CSF (4.16/0.50 s) [33–35], relative water contents in GM (0.78), WM (0.65) and CSF (1.0) [36], and T1 and T2 of Glu in the GM (1.27/0.16 s) and WM (1.17/0.17 s) [36,37], respectively. Thus, corrected metabolite concentrations are given in mmol per Kg. Because creatine levels was found to be predicted by CB use [20] we did not normalize other metabolites to creatine (see Newman et al. [27] for voxel placement and analysis details). Please see supplementary material for details about the quality of the taurine measure.

### Experiment 2: Urine metabolomics

#### Participants

A separate group of Twenty-seven cannabis users were included in the analysis (see Table 1 for demographic information). Subjects were recruited by local advertisements. After detailed description of the study, written and verbal informed consent was obtained from each participant. Subjects were asked to refrain from alcohol or CB use the day prior to the study. This study was carried out in accordance with the recommendations of and approved by Indiana University’s Institutional Review Board for the protection of human subjects. All subjects gave written informed consent in accordance with the *Declaration of Helsinki*.

**Table 1:** Experiment 2 participants.

	CB Users
n	27
#Males	10
Age	21.3±2.4 (18–30 years)
Age of CB initiation	17.9±1.9 years
Frequency of CB use	Alone use: 8/7/10/2 use daily/weekly/monthly/never Group use: 9/12/6 use daily/weekly/monthly
WASI	118.2±10.5

Note: WASI = Wechsler Abbreviated Scale of Intelligence; CB = cannabis.

The exclusion criteria included: younger than 18 years or older than 40; presence of any neurological disorder; history of head trauma with loss of consciousness greater than ten minutes; learning disability; diagnosis of psychosis; use of illicit drugs (other than CB); and alcohol dependence.

Participants completed a battery of assessments including the Diagnostic Interview for Anxiety, Mood, and Obsessive-Compulsive and Related Neuropsychiatric Disorders (DIAMOND) which is a semi-structured diagnostic interview for DSM-5 psychiatric disorders [38]; the Wechsler Abbreviated Scale of Intelligence (WASI [31]); the Alcohol Use Disorders Identification Test (AUDIT [39]) and the Cannabis Use Disorders Identification Test (CUDIT [40]). Twelve participants had depression and 3 had anxiety at time of participation.

Participants provided information regarding their CB use. They indicated the frequency of their use (e.g., daily, weekly, monthly or less frequent). In addition, participants indicated how often they used alone or with others as well as how the method of use (e.g., pipes, joints, vaping oil, edibles). The characteristics of the sample were similar to the CB user group in Expt 1 in gender, age, age of CB initiation, frequency of use and WASI score.

#### Urine metabolite extraction

Urine aliquots (50 μL) of urine were treated with ice-cold methanol (200 μL). Samples were stirred continuously for 30 minutes and then centrifuged at 3000 rpm for 10 minutes at 4°C. Supernatants were placed in a new glass tube and evaporated to dryness under N_2_. Residues were resuspended in 0.1% formic acid (500 μL),

### LC-MS/MS analysis

An aliquot (10 μL) of each sample was loaded onto a Phenomenex 2.1 x 100 mm, 2.7 μm Luna Omega, 80 Å reverse-phase column (Phenomenex, Torrance,CA). Chromatography used the mobile phases, A: ddH_2_O with 0.1% formic acid and B: acetonitrile/0.1% formic acid. Initially, the column was equilibrated with A. After injection of the sample, metabolites were eluted with a 5 minute linear gradient of B (2–50%), then a 1 minute linear gradient of 50–98% B until 6.0 min followed by a 1 minute hold at 98% B, and finally re-equilibration at initial conditions for 3 minutes. An Exion UHPLC (Sciex, Toronto, Ontario) provided a flow rate of 500 μL/minute. A SCIEX 5600 Triple-Tof mass spectrometer (SCIEX, Toronto, Canada) was used to analyze the metabolite profiles. The IonSpray voltages for positive and negative modes were +5000 and -4500 V, respectively, and the declustering potential was +/- 80 V. IonSpray GS1/GS2 and curtain gases were set at 40 psi and 25 psi, respectively. The interface heater temperature was 400°C. Eluted compounds in individual samples were subjected to successive 250 msec time-of-flight survey scans over the mass-to-charge (*m/z*) range from 50–1000 to capture sample metabolite ion elution profiles. Pooled samples, containing 20 μL of each experiment specimen, were injected every tenth analysis to assess instrument stability using the same data collection protocol. In addition, separate aliquots (20 μL) of the pooled samples were subjected to a modified data collection protocol–first, a 100 msec time-of-flight survey scan from *m/z* 50–1000 to determine the top eight most intense ions for MSMS analysis followed by 50 msec product ion time-of-flight scans to obtain the tandem mass spectra of the selected parent ions over the range from *m/z* 50–1000 using a collision energy spread of 15 eV with a set collision point of 35 eV. Spectra were centroided and de-isotoped by Sciex Analyst software, version 1.71.

### Data analysis and metabolite identification

LC-MS data were processed using MS-DIAL version 4.48 [41] to determine ion features (peaks) occurring across all samples, their peak areas and retention times. Versions 15 of the Public MSMS positive and negative databases were used for ion feature annotations. Identifications of metabolites were also assessed against the IROA 600 standard compound library (IROA Technologies, Sea Girt, NJ) and verified by evaluating product ion spectra of each target using PeakView 2.0 Software (SCIEX, Toronto, Ontario). In addition, ion chromatograms for targeted compounds of interest, taurine and 11-nor-9-carboxy-Δ9-tetrahydrocannabinol β-glucuronide, were extracted from the profile data to capture peak areas for correlation studies using PeakView 2.0 Software suite.

#### Statistical analysis

Between subjects ANOVAs and correlation analyses were performed using SAS version 9.4. Moderation analyses were performed using the Process module in SPSS [42].

## Results

### Experiment 1

After data cleaning, data from 26 CB users and 24 non-users were analyzed. We first performed a one-way ANOVA to examine group differences. There were no differences in taurine levels [F<1; CB users: M = 0.91±0.3; non-users: M = 0.96±0.3]. As previously reported for glutamate [20], there were no group differences [F<1; CB users: M = 5.41±0.4; non-users: M = 5.29±0.4].

**Fig 1:**
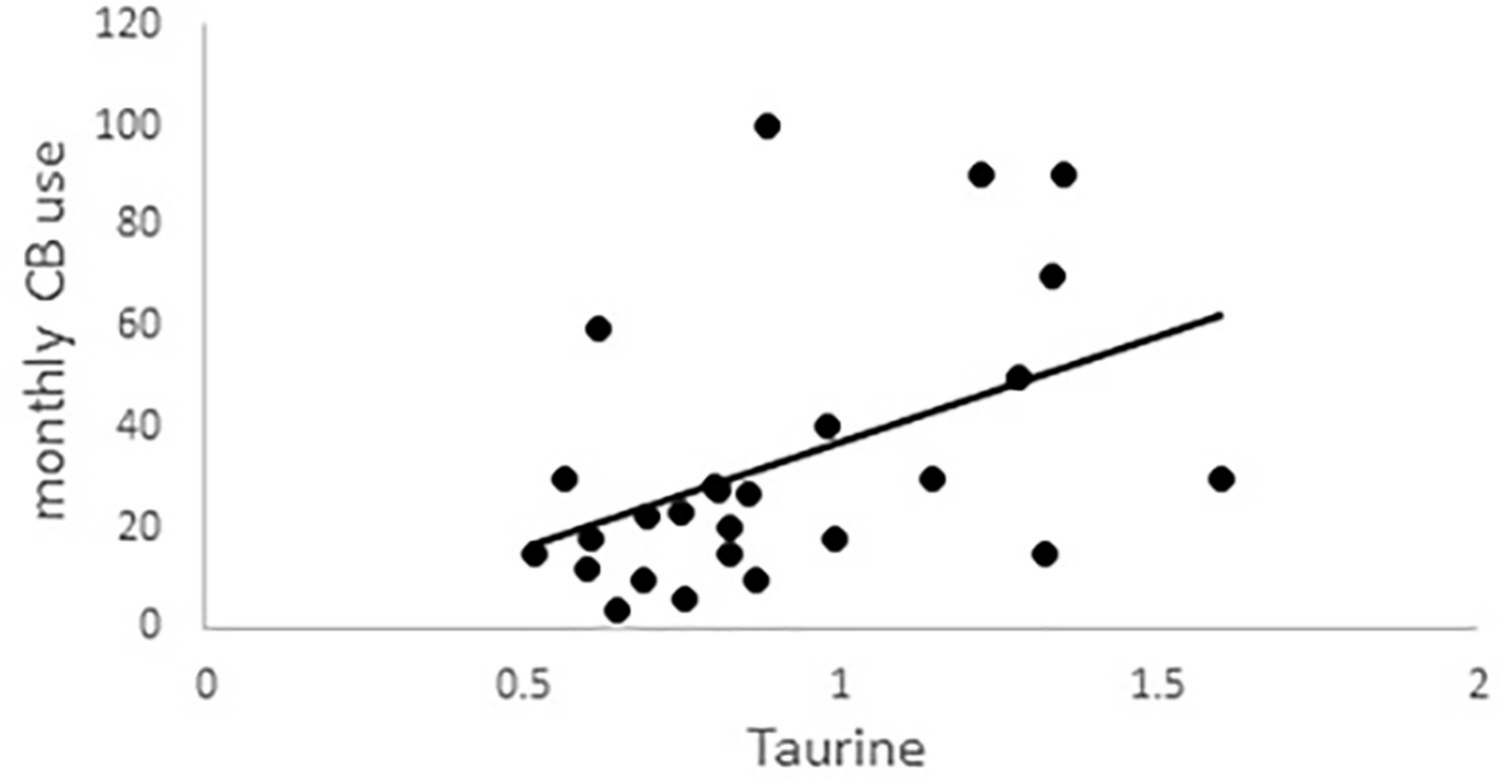
Expt 1 results. Correlation between taurine and monthly CB use in the CB user group.

A correlation analysis was then performed to examine the relationship between taurine and glutamate. This analysis was performed separately for the CB users and non-users. Taurine was found to be significantly correlated with glutamate in both groups [non-user group: r = 0.63, p = 0.001; CB group: r = 0.45, p = 0.022]. We then performed a correlational analysis to examine the relationship between taurine and monthly CB use, only for the CB user group. Taurine was also found to be correlated with monthly CB use (Fig 1; r = 0.48, p = 0.014).

To further examine the relationship between CB use and taurine a moderation model was analyzed. Taurine has been found to be lower in individuals with higher body fat [43]. Because cannabinoids are absorbed in body fat [44] and released over time, we examined whether body composition might moderate any associations between CB use and taurine levels. A moderation model revealed significant effects of monthly CB use and BMI [F(3,22) = 4.85, p = 0.0095, R^2^ = 0.4] with the effects of monthly CB use and BMI being significant (see Table 2). The interaction was marginally significant (see Table 2) with the effect of CB use having a significant effect at smaller BMIs (see Table 3).

**Table 2:** Model (Expt1).

	coeff	se	t	p	LLCI	ULCI
constant	-0.2502	0.384	-0.6516	0.5214	-1.0467	0.5462
Monthly CB use	0.0249	0.0099	2.5153	***0*.*0197***	0.0044	0.0453
BMI	0.0403	0.0153	2.64	***0*.*015***	0.0086	0.072
Interaction	-0.0008	0.0004	-2.0278	***0*.*0549***	-0.0017	0

**Table 3:** Conditional effects monthly CB use at values of the moderator(s).

BMI	Effect	se	t	p	LLCI	ULCI
19.884	0.0085	0.0024	3.5	***0*.*002***	0.0035	0.0136
23.23	0.0058	0.0018	3.1997	***0*.*0041***	0.002	0.0095
30.1684	0.0001	0.0031	0.0216	0.983	-0.0063	0.0064

### Experiment 2

**Fig 2:**
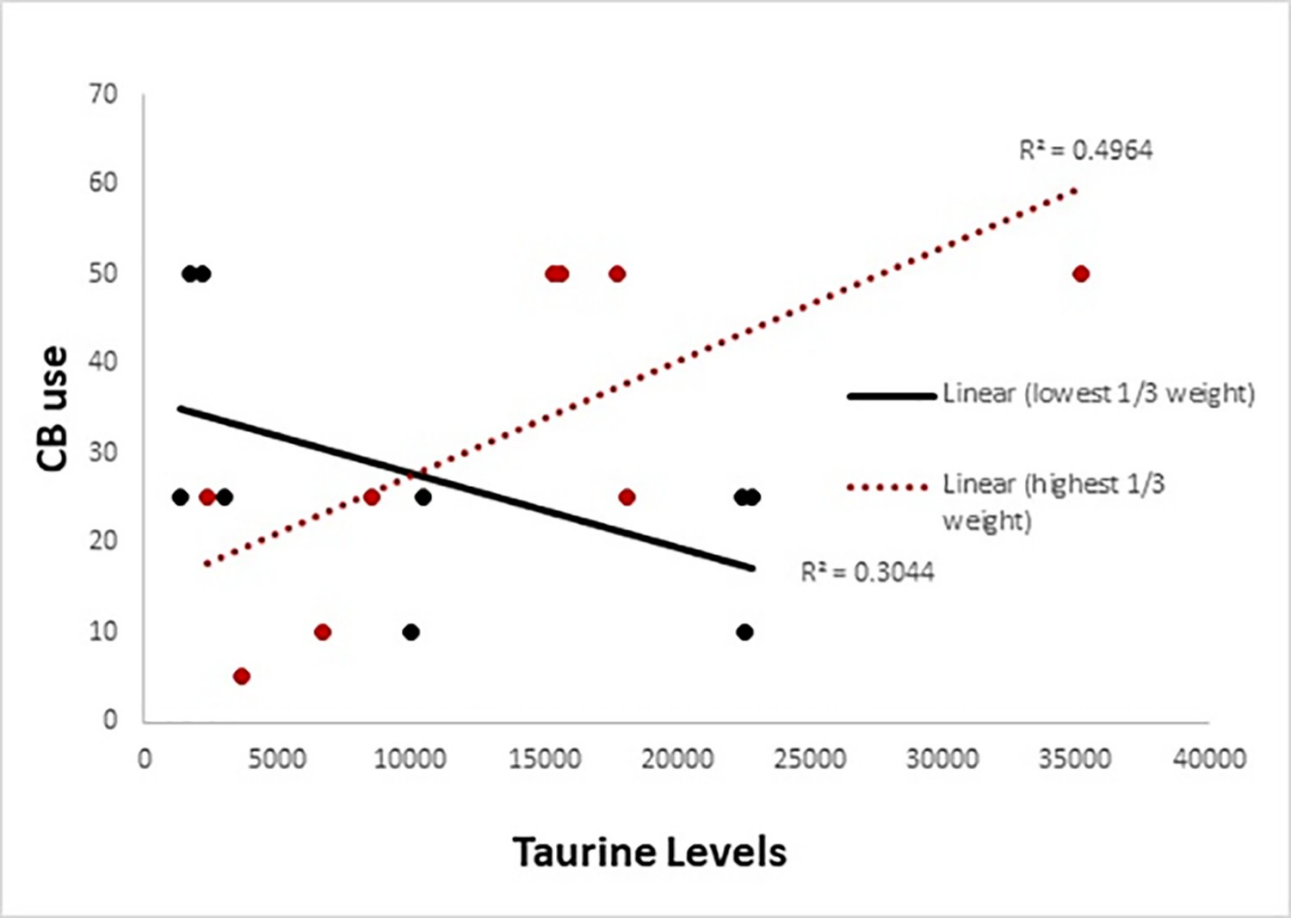
Expt 2 results. For demonstration purposes only in order to visualize the interaction for Expt 2 (metabolomics analysis), participants were ranked into thirds based on weight. The red line depicts the relationship between CB use and taurine for the participants with the highest weight and the black line depicts those with the lowest weight.

The mean intensity and area under the curve for taurine was 11217.8±10157.1 and 109987.5±101043, respectively. A similar moderation model was performed for the metabolomics analysis to examine the relationship between monthly CB use and taurine moderated by body weight. The moderation model was significant [F(3,23) = 2.93, p = 0.055, R2 = 0.53] with the frequency of CB use, weight and interaction being significant (see Table 4 and Fig 2). The interaction revealed that there was an association between CB use and taurine only for individuals with higher weight (see Table 5).

**Table 4:** Model.

	coeff	se	t	p	LLCI	ULCI
constant	-248.3239	127.9294	-1.9411	0.0646	512.977	16.3295
Frequency CB use	40.8264	18.622	2.1924	***0*.*0387***	2.3022	79.3507
Weight	2.1734	0.8538	2.5455	***0*.*0181***	0.407	3.9397
Interaction	-0.296	0.1227	-2.4116	***0*.*0243***	-0.5499	-0.0421

**Table 5:** Conditional effects monthly CB use at values of the moderator(s).

Weight	Effect	se	t	p	LLCI	ULCI
122.4	4.5945	4.3846	1.0479	0.3056	-4.4761	13.6652
145	-2.0953	2.849	-0.7355	0.4695	-7.9892	3.7985
165	-8.0156	3.3352	-2.4034	**0.0247**	-14.9152	-1.116

## Discussion

The goal of this study was to examine the relationship between taurine and CB use using two methods–MRS, and metabolomics analysis of urine–and two groups of participants. Both approaches showed that frequency of CB use predicted taurine levels and that the relationship was moderated by body composition (BMI or weight). Given taurine’s neuroprotective effects and its abundance in the brain, the results demonstrate the need to better understand the mechanism(s) that underlie(s) this relationship.

There are few studies that have linked taurine to CB. Hinckley and colleagues [6], when examining plasma, found that taurine levels were positively correlated with THC-COOH levels, a metabolite of THC. The results presented in the current study corroborate those previous results by showing that for the MRS analysis taurine measured in the dACC was positively correlated with frequency of CB use. The urine analysis also showed a relationship between taurine levels and CB use that was moderated by body weight. Interestingly, while an association between taurine and CB use was observed, there was no significant difference in taurine levels for CB users and non-users. A similar finding was observed for glutamate in a previous study in which a relationship between glutamate and monthly CB use moderated by sex was observed even though no group differences were observed [27]. The effect of monthly use supports previous work that suggests a blunted response to acute administration for heavy users [45] as heavy users have larger baseline differences compared to non-users.

Taurine is an inhibitory amino acid that protects cells [46,47], including from toxic amounts of glutamate [48]. In fact, taurine release has been found to be evoked by an agonist to glutamate receptors, N-methyl-D-aspartate and kainite [49]. The results of the current MRS study are in line with this view and revealed a positive correlation between taurine and glutamate. Interestingly, THC has been found in animal models to modulate glutamatergic neurotransmission and concentrations. The CB1 receptor is highly expressed on glutamatergic neurons and inhibit synaptic transmission. Brown and colleagues [50] reported that THC reduces the release and uptake of glutamate in a dose-dependent manner in rat striatal slices. Straiker and Mackie [51] showed CB receptor-mediated reduction in glutamate transmission in mice. Findings in human studies have been mixed. Some previous MRS studies have shown that CB use is associated with decreases in glutamate [52,53]. Others failed to find a direct relationship between glutamate and CB use [27]. While others have reported increases in glutamate in CB users [26,54]. One issue is the variation in the location in which glutamate is measured. In the current study, no statistically significant difference was observed between groups for either glutamate or taurine in the dACC. However, Mason and colleagues [54] found significant increases in glutamate after acute administration in the striatum but not in the ACC. Another issue with using MRS that may complicate the direct correlation between human and preclinical studies is that MRS measures levels in both intra- and extracellular space while preclinical studies do not. Taurine may offer a better measure to explore using MRS especially given its relationship to glutamate.

Taurine, like CB, has effects throughout the body. Taurine is present and has numerous roles in the periphery reducing oxidative stress related to kidney disease [55] and diabetes [56]. The results presented in the current study demonstrate that both taurine measured in brain and the periphery is associated with CB use. Given taurine’s role in mediating the effects of oxidative stress, it is not only neuroprotective but cell protective, generally. The link between taurine and CB may provide clues as to the mechanism that underlies some of the medicinal effects of CB.

A significant finding of the current study is that body composition moderated the effect of CB on taurine levels. The effects were different for the MRS and metabolomics studies. For MRS the effects were larger for smaller individuals and for the metabolomics analysis, the effect was smaller for smaller individuals. While the mechanisms that underlie this difference is not clear, we hypothesize that differences are due to the rapid uptake of THC by body fat [57]; it is stored in body fat and is released slowly. Therefore, body composition affects how much THC is in plasma and reaches the brain. More research in humans is necessary to better characterize the effect of body composition on the relationship between THC and taurine.

There are a number of limitations of the current study. Both experiments have a small sample size. In addition to future studies having a larger sample size, it is also necessary to perform more controlled studies in which diet is better controlled. Taurine is influenced by diet [58], particularly energy drinks. In the current study, self-reported CB use was obtained. Future work should explore the relationship between THC and CBD separately. It should also be noted that neither BMI nor weight provides an accurate measure of body fat; future studies should use a better measure. Finally, given our previous work showing sex differences in the effect of CB use [27,59] and potential differences in metabolism [60], examining sex differences in the association between CB use and taurine is warranted.

Finally, there are some considerations related to using MRS to measure glutamate and taurine. There are concerns regarding the ability to separate glutamate and glutamine. However, a recent study demonstrated that the glutamate can be estimated with good accuracy at 3T while glutamine is not reliably estimated [61]. Taurine is difficult to measure because of its overlap with other macromolecules, making its signal noisy. This was observed in the current study as well (see S2 and S3 Figs in S1 File and S1 Table in S1 File). However, as shown in the supplementary materials (see S2 Fig in S1 File), the contribution of taurine is appreciable and easily measured with LCModel.

## Conclusions

This study was designed to demonstrate a potential relationship between CB use and taurine. The results presented provide a foundation for future studies to examine this relationship. The mechanism by which CB modulates taurine levels is unclear and to the authors’ knowledge, there are no studies that characterize the relationship between taurine and CB. Interestingly n-arachidonoyl taurine, a fatty acid, has been linked to the endocannabinoid system^62^. Future research examining these relationships is necessary.

## Supporting information

S1 File Figures and tables that demonstrate the quality of the MRS taurine measure. (DOCX)
