# Supplementary material for: The relationship between cannabis use and taurine: A MRS and metabolomics study
Source: PLoS One. 2022 Jun 2;17(6):e0269280. doi: 10.1371/journal.pone.0269280 (PMC9162360; doi:10.1371/journal.pone.0269280)
Supplement: S1 File — (DOCX) [file pone.0269280.s001.docx]

**
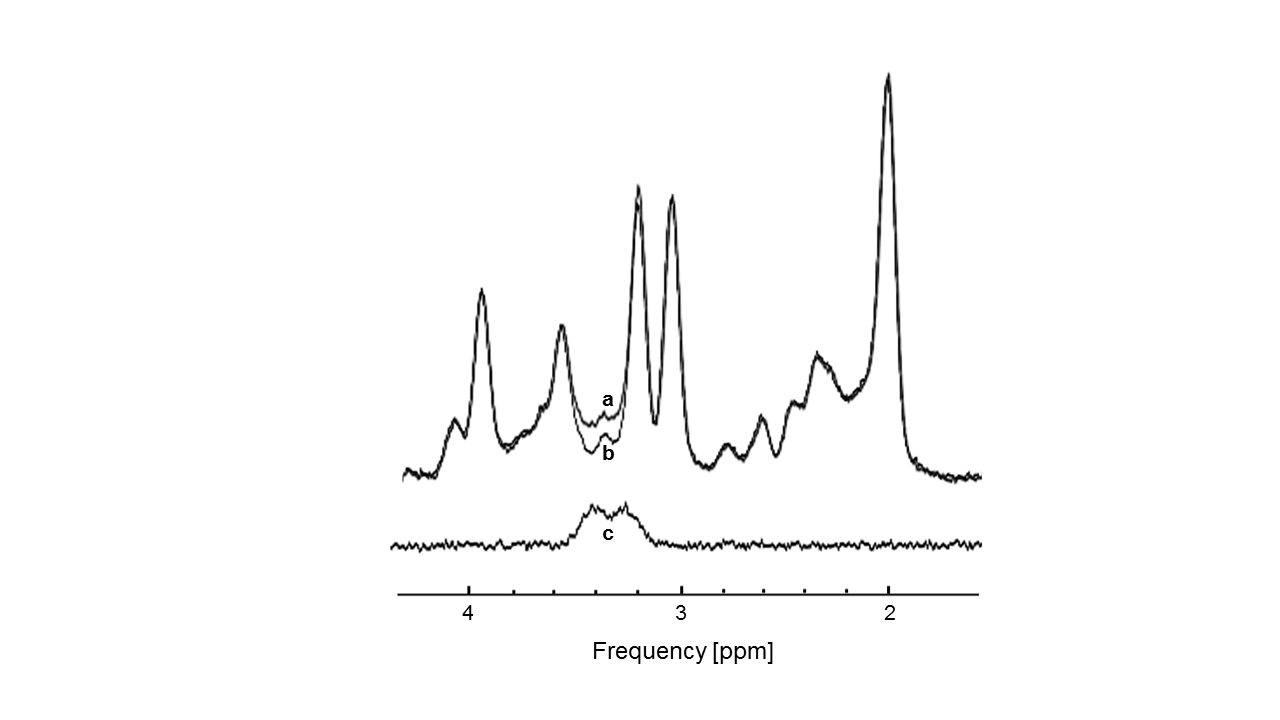
Supplementary Materials**

Figure S1:  Synthetic data demonstrating the taurine peak and the non-negligible contribution of taurine to the spectra. (a) is the synthesized spectrum as described in a recent paper (H. Cheng, et al., 2020); (b) is the synthetic spectra without Tau; (c) is the difference.


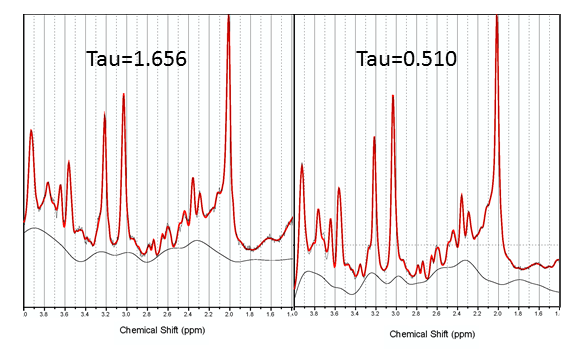


Figure S2:  Two representative spectra one from the highest and lowest taurine concentrations.

**Table S1: Concentration of metabolites exported from LCModel for a representative subject**

| Metabolite | Concentration (I.U.) | CRLB | Ratio to tCr |
| --- | --- | --- | --- |
| Asp | 1.977 | 12% | 0.404 |
| Creatine | 2.727 | 6% | 0.558 |
| Glutamine | 1.068 | 16% | 0.218 |
| Glutamate | 5.665 | 7% | 1.159 |
| Choline | 1.237 | 2% | 0.253 |
| Myo-inositol | 5.466 | 4% | 1.118 |
| NAA+NAAG | 6.158 | 2% | 1.260 |
| phosphocreatine | 2.162 | 7% | 0.442 |
| Taurine | 1.071 | 24% | 0.219 |


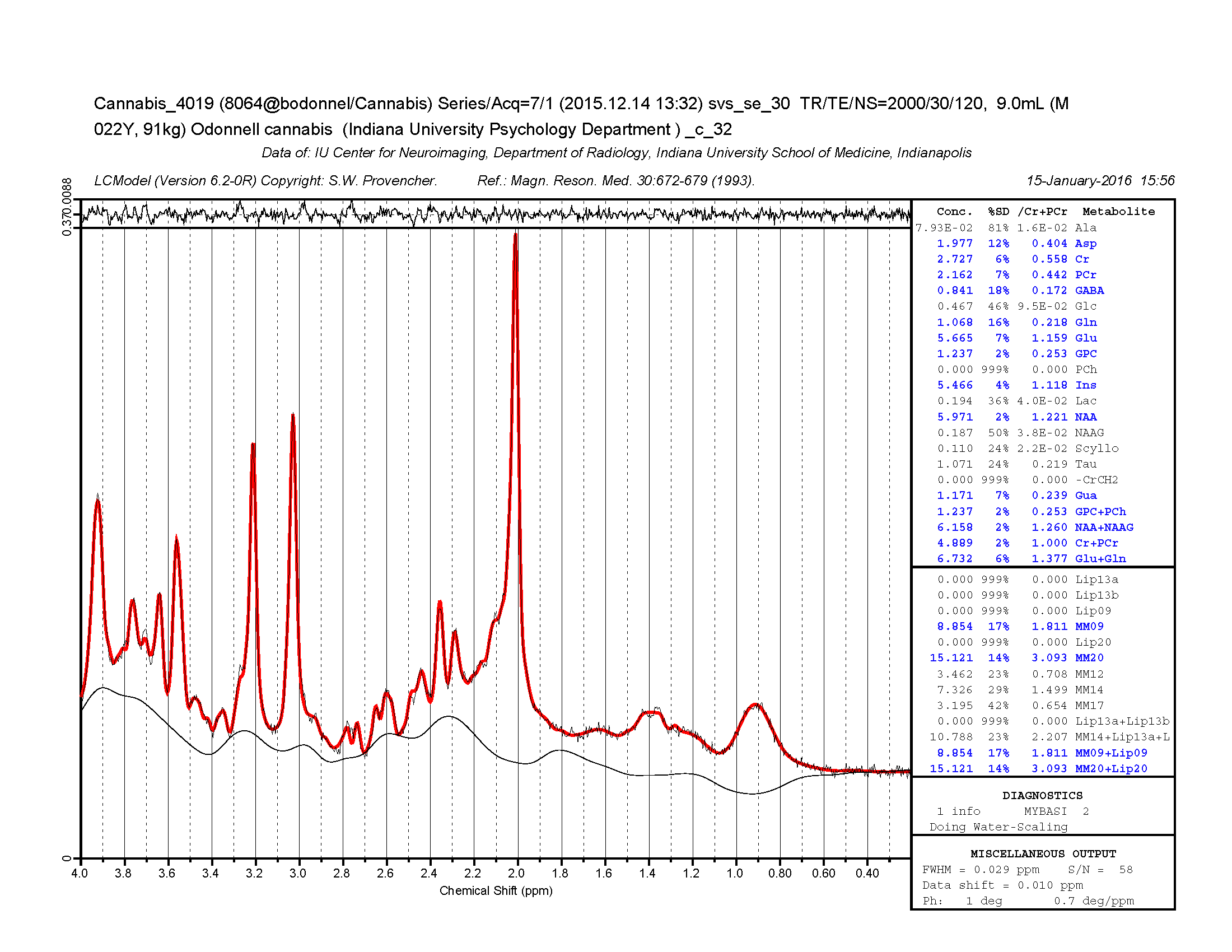


The taurine peak is around 3.4 ppm, but overlapped with choline near 3.2 ppm and myo-inositol near 3.6 ppm. Therefore, we only selected a narrow band of 3.3-3.5 ppm (shaded in light blue) to show the fitting of taurine by LCModel (black: raw spectrum, red: fitted spectrum). The residual of fitting is displayed at the top.


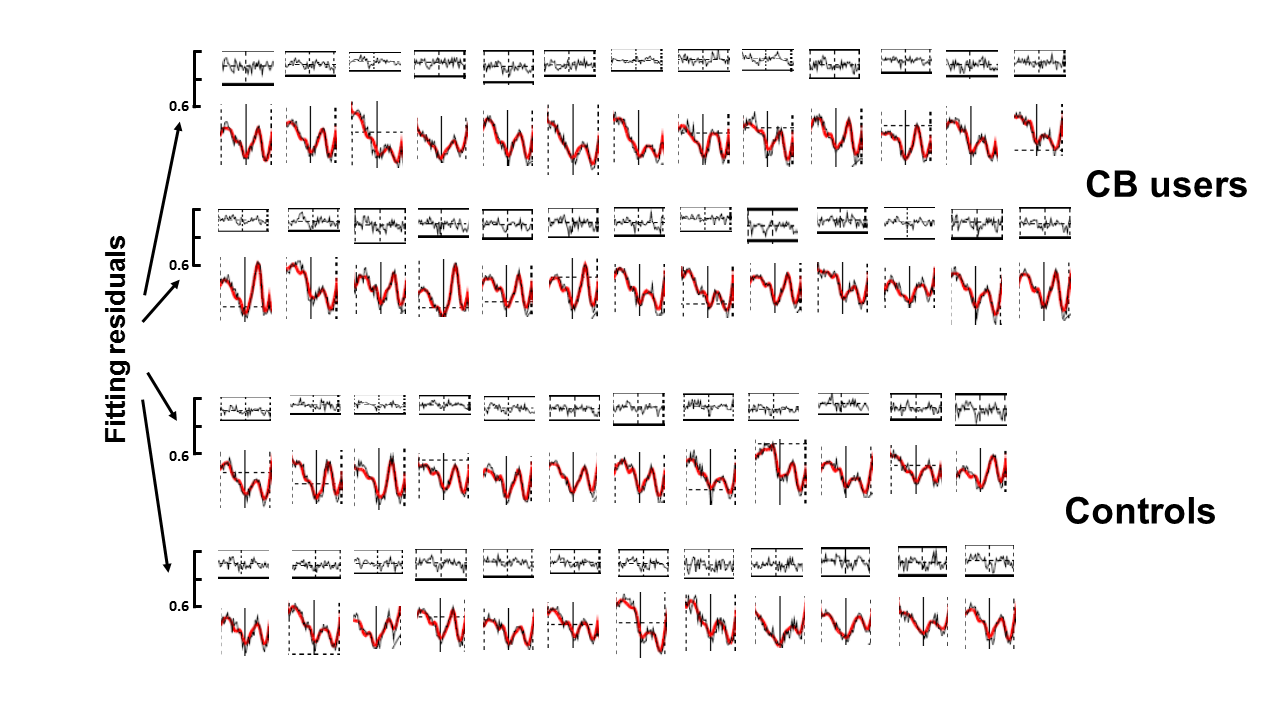


Figure S3: Each participant’s spectra.
